# Structural, Electrical and Electrochemical Properties of Glycerolized Biopolymers Based on Chitosan (CS): Methylcellulose (MC) for Energy Storage Application

**DOI:** 10.3390/polym13081183

**Published:** 2021-04-07

**Authors:** Shujahadeen B. Aziz, Ahmad S. F. M. Asnawi, Mohd Fakhrul Zamani Kadir, Saad M. Alshehri, Tansir Ahamad, Yuhanees M. Yusof, Jihad M. Hadi

**Affiliations:** 1 Hameed Majid Advanced Polymeric Materials Research Lab., Physics Department, College of Science, University of Sulaimani, Qlyasan Street, Kurdistan Regional Government, Sulaimani 46001, Iraq; 2 Department of Civil Engineering, College of Engineering, Komar University of Science and Technology, Kurdistan Regional Government, Sulaimani 46001, Iraq; 3 Chemical Engineering Section, Universiti Kuala Lumpur Malaysian Institute of Chemical & Bioengineering Technology (UniKL MICET), Alor Gajah 78000, Malaysia; asyafiq.asnawi@s.unikl.edu.my (A.S.F.M.A.); yuhanees@unikl.edu.my (Y.M.Y.); 4 Centre for Foundation Studies in Science, University of Malaya, Kuala Lumpur 50603, Malaysia; mfzkadir@um.edu.my; 5 Department of Chemistry, King Saud University, P.O. Box 2455, Riyadh 11451, Saudi Arabia; alshehri@ksu.edu.sa (S.M.A.); tahamed@ksu.edu.sa (T.A.); 6 Department of Medical Laboratory of Science, College of Health Sciences, University of Human Development, Kurdistan Regional Government, Sulaimani 46001, Iraq; jihad.chemist@gmail.com

**Keywords:** chitosan, methylcellulose, ammonium thiocyanate, glycerol, ionic transport parameters, TNM and LSV, CV and EDLC

## Abstract

In this work, a pair of biopolymer materials has been used to prepare high ion-conducting electrolytes for energy storage application (ESA). The chitosan:methylcellulose (CS:MC) blend was selected as a host for the ammonium thiocyanate NH_4_SCN dopant salt. Three different concentrations of glycerol was successfully incorporated as a plasticizer into the CS–MC–NH_4_SCN electrolyte system. The structural, electrical, and ion transport properties were investigated. The highest conductivity of 2.29 × 10^−4^ S cm^−1^ is recorded for the electrolyte incorporated 42 wt.% of plasticizer. The complexation and interaction of polymer electrolyte components are studied using the FTIR spectra. The deconvolution (DVN) of FTIR peaks as a sensitive method was used to calculate ion transport parameters. The percentage of free ions is found to influence the transport parameters of number density (*n*), ionic mobility (*µ*), and diffusion coefficient (*D*). All electrolytes in this work obey the non-Debye behavior. The highest conductivity electrolyte exhibits the dominancy of ions, where the ionic transference number, *t_ion_* value of (0.976) is near to infinity with a voltage of breakdown of 2.11 V. The fabricated electrochemical double-layer capacitor (EDLC) achieves the highest specific capacitance, *C_s_* of 98.08 F/g at 10 mV/s by using the cyclic voltammetry (CV) technique.

## 1. Introduction

The approaches towards the application of natural solid polymer electrolytes in electrochemical devices such as proton batteries and electrochemical double-layer capacitors (EDLCs) has received attention in this era of technology [1]. Several studies showed the potential of natural solid polymer electrolytes for device applications due to their superior mechanical and chemical performance [2,3,4,5,6]. Due to the ability to achieve a high specific capacitance, power density as well as endurance, the EDLC has become a suitable replacement candidate to the other capacitors [7,8,9]. Since the storage mechanism follows the non-Faradaic process, an EDLC only involves the accumulation of ions that is caused by the fast reversible charges carrier’s adsorption at the electrode/electrolyte interfaces [10]. Due to the characteristics such as a large surface area, high porosity, and being non-expensive, activated carbon is one of the most used materials for the electrode in EDLCs [11,12].

In addition, the advantage of using natural polymers in the electrochemical study, especially in the fabrication of EDLCs, is that it could prevent the impact of environmental pollution which might be due to the conventional batteries [13,14]. Chitosan (CS) is the widespread category of polymers used in the preparation of polymer electrolytes because it has biodegradable and biocompatible properties [15,16,17]. CS is produced from the deacetylation process of chitin and because of its chemical composition, the inclusion of (OH and NH_2_) in its backbone structure, CS can be a strong ionic conductor [18,19,20]. Moreover, due to several properties such as good thermal and mechanical strength as well as biocompatibility, methylcellulose (MC) is also usually used as a polymer host component in polymer electrolyte synthesis [21]. MC is a derivative of cellulose that has the amphiphilic ability due to the various hydrophilic carboxyl and hydrophobic polysaccharides present in the chemical backbone. Both polymers have been found to be full of oxygen containing functional groups with lone pair electrons that are potential carriers for ionic conduction [22,23].

In conjunction with preventing environmental pollution which could be due to the usage of lithium-based salts, many studies proved that ammonium-based salts such as NH_4_SCN [6,24], NH_4_NO_3_ [25,26], NH_4_Br [27,28], and NH_4_F [29], have promising polymer electrolyte characteristics with a comparable dissociation of ions [30]. Besides, the presence of NH_4_^+^ and H^+^ in the ammonium-based electrolyte could achieve a high ionic conductivity [31]. The low lattice energy of NH_4_SCN (605 kJ/mol) makes the electrolyte need a lesser amount of energy to break the ionic bonds, which is crucial in optimizing the conductivity value of the electrolytes [32,33]. Furthermore, the dissociation of the ions can be developed with the incorporation of a plasticizer which also can improve the ionic conductivity, amorphousness, and thermal properties of the electrolyte that is important for the high performance of future energy device applications [34,35,36]. Many studies in the literature formulated the polymer electrolytes with the addition of various glycerol concentrations as the function to plasticize the respective systems [37,38,39]. Thus, this work describes the effect of different glycerol concentrations on the CS–MC–NH_4_SCN electrolyte system and consequently, electrolytes with the most satisfactory performance will be employed in the fabrication of EDLCs.

## 2. Methodology

### 2.1. Sample Preparation

The preparation of the polymer blend host in this work involved chitosan (CS) and methylcellulose (MC) which was directly used as purchased from Sigma–Aldrich (Darmstadt, Germany). Under an ambient condition, CS (70 wt.%) and MC (30 wt.%) were firstly dissolved separately in 1% acetic acid (40 mL) for 3 h. The CS and MC solutions were mixed and stirred to obtain a homogeneous CS–MC blend solution. To prepare a polymer electrolyte, a fixed amount of ammonium thiocyanate (NH_4_SCN) (40 wt.%) was added to the CS–MC blend solution. Consequently, three different concentrations of glycerol plasticizer (14, 28, and 42 wt.%) were added separately into the CS–MC–NH4SCN polymer electrolyte solution where the samples were coded as CSMCD1, CSMCD2, and CSMCD3, respectively. Once the glycerolized CS–MC–NH_4_SCN solutions reached a homogeneous state, they were cast into plastic Petri dishes and then left to dry for several days. The thickness of the membranes were in the range of 121–123 µm. The samples were kept in a desiccator prior to the characterizations.

### 2.2. Impedance Study

One of the significant characteristics that need to be studied is the impedance properties of polymer electrolytes. This analysis was conducted using the HIOKI 3532-50 LCR HiTESTER(HIOKI, Nagano, Japan) at 50 Hz to 5 MHz under room temperature. By taking the values of bulk resistance (*R_b_*) from Nyquist plots as well as the surface area, *A* and thickness, *d* of the electrolytes, the ionic conductivity, *σ* values of the electrolytes can be determined using the following relation:

(1) $$\sigma =\frac{d}{A}\times \frac{1}{R_{b}}$$

### 2.3. FTIR Study

Furthermore, the Fourier transform infrared (FTIR) spectroscopy analysis was also carried out to investigate the interaction and complexation among the polymers, salt, and plasticizer. The FTIR analysis in this work was employed using a Spotlight 400 Perkin–Elmer spectrometer (Perkin Elmer, Melville, NY, United States) with 1 cm^−1^ resolution in the range of 500 to 4000 cm^−1^. To further support the ionic conductivity studies, the ionic transport parameters; number density (*n*), ionic mobility (*µ*), and diffusion coefficient (*D*) based on the percentage of free ions were identified. The deconvolution DVN method via the Gaussian–Lorentzian function was used to extract overlapping peaks as well as correct the baseline of the curves. The percentage of free ions and the transport parameters can be determined using the following equations [28,40,41].

(2) $$Free\;ions\;\%=\frac{A_{f}}{A_{f}+A_{c}+A_{g}}\times 100$$

(3) $$n=\frac{M\times N_{A}}{V_{Total}}\times \left(free\;ion\;\%\right)$$

(4) $$\mu =\frac{\sigma }{ne}$$

(5) $$D=\frac{\mu kT}{e}$$

where *A_f_* denotes the area below the peak in terms of the free ion’s region, *A_c_* stands for the area under the peak in terms of the contact ion region, and *A_g_* represents the area below the peak in terms of the ion aggregates region. *M* represents the number of moles of glycerol, *N_A_* is Avogadro’s number and *V_Total_* is the total volume of the polymer electrolytes. *k* and *e* are the Boltzmann constant and elementary charge, respectively, while *T* represents the temperature in Kelvin.

### 2.4. Transference Number Measurement (TNM) and Linear Sweep Voltammetry (LSV) Measurements

The ion dominancy within the electrolyte, based on the ionic (*t_ion_*) and electronic (*t_elec_*) transference number values, can be studied using the transference number measurement (TNM). This analysis was performed using a V&A Instrument DP3003 digital DC power supply (V & A Instrument, Shanghai, China) at 0.20 V operating voltage. Besides, the linear sweep voltammetry (LSV) was employed to measure the breakdown voltage of an electrolyte which was carried out using a Digi-IVY DY2300 potentiostat (Neware, Shenzhen, China) with a 10 mV/s scan rate. The stainless steel (SS) electrodes were used with only the highest conducting electrolyte that was involved for these analyses and the cell arrangement was SS|electrolyte|SS.

### 2.5. EDLC Fabrication and Characterization

Based on our previous work on the fabrication of EDLCs, the preparation of activated carbon electrodes was well-explained [42,43]. The specific capacitance, *C_s_* value for the EDLC was determined using the cyclic voltammetry (CV) that was carried out on a Digi-IVY DY2300 potentiostat at the sweep rates of 10 to 100 mV/s. The mass of the coated AC electrode on the aluminum foil was 0.00243 g and its thickness was 0.02 cm. The area of the electrode was 2.01 cm^2^.

## 3. Results and Discussion

### 3.1. Impedance and Circuit Modeling Analysis

**Figure 1:**
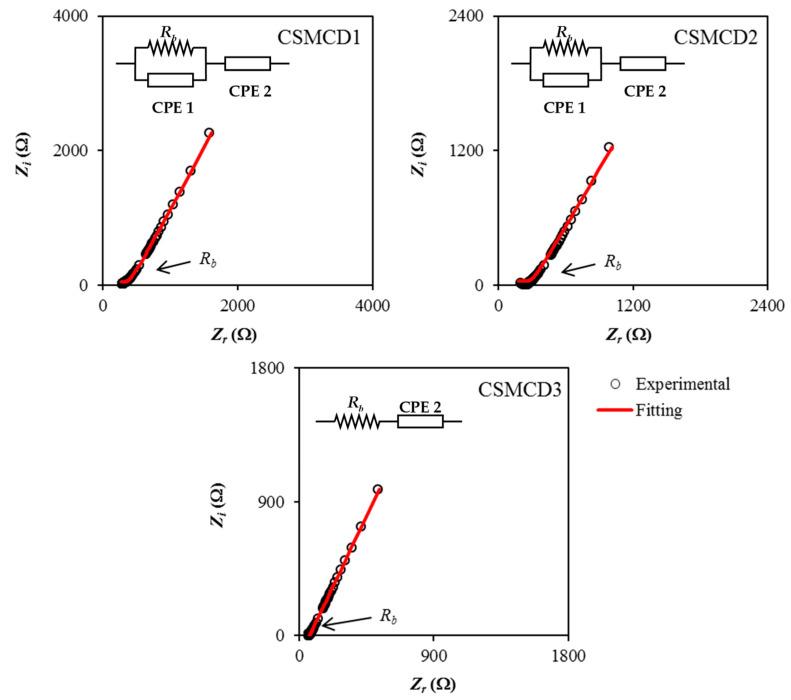
Impedance plots for the electrolytes.

The electrochemical impedance spectroscopy (EIS) plots for the electrolytes at room temperature are shown in Figure 1. EIS is beneficial to determine the impedance properties of the films. It can be observed that the plot consists of a curved portion of the data points at high frequency for the CSMCD1 and CSMCD2 systems which represent a parallel combination of a constant phase element (CPE) and a resistor, where CPE indicates the immobile polarized polymer chains in the alternating field while the resistor is for the migration of ions [44,45]. For the CSMCD3 system, only a spike can be evidenced at 42 wt.% of glycerol. Another obvious region that can be observed is a non-vertical spike (less than 90°) at a low frequency that is associated with the electrodes blocking effects which also suggests the in-homogeneity surface at the electrolyte-electrode interface. This phenomenon leads to the inability of mobile ions to penetrate the electrode but it will form an electrical double layer near to the surface of each electrode [46].

Based on Figure 1, the presence of a curved portion of the data points is noticed to disappear with the increase in glycerol concentration to 42 wt.% where the bulk resistance, *R_b_* value is reduced (see Table 1). This situation is caused by the resistive element in the electrolyte that decreases and can be estimated from the interception of *R_b_* to the real impedance (*Z_r_*) axis [47]. For CSMCD1 and CSMCD2, the *R_b_* value is obtained from the interception of a semicircle and the *Z_r_*, while the interception of the inclined spike and *Z_r_* axis shows the *R_b_* value in CSMCD3. The highest conductivity value obtained in this work is 2.29 × 10^−4^ S cm^−1^ by the CSMCD3 electrolyte which was found to be compatible with the application of electrochemical devices that normally applied an electrolyte with a conductivity of ~10^−3^ to 10^−5^ S cm^−1^. The conductivity is higher than the CS–MC polymer blend host that was reported at 3.35 × 10^−9^ S cm^−1^ [48]. Researchers also found that these ranges of conductivity values are beneficial to be applied in the energy devices [27,49,50,51,52]. Hence, the CSMCD3 electrolyte could promise a good performance of energy devices.

Further understanding of the electrical properties of each electrolyte can be inspected by means of the electrical equivalent circuits (EECs) as shown in the impedance plots (Figure 1). From the EECs modeling, the values of circuit elements can be identified where the expression of CPE impedance (*Z_CPE_*) is as follows [53,54,55]:

(6) $$Z_{CPE}=\frac{1}{C\omega^{p}}\left[\cos\left(\frac{\pi p}{2}\right)-i\sin\left(\frac{\pi p}{2}\right)\right]$$

In Equation (6), *C* and *ω* represent the CPE capacitance and the angular frequency, respectively, while *p* is the deviation of the plot from the axis. The real impedance, *Z_r_* and imaginary impedance, *Z_i_* of the plots that consist of both spike and semicircle (CSMCD1 and CSMCD2) can be calculated using the equations below:

(7) $$Z_{r}=\frac{R_{b}C_{1}\omega^{p_{1}}\cos\left(\frac{\pi p_{1}}{2}\right)+R_{b}}{2R_{b}C_{1}\omega^{p_{1}}\cos\left(\frac{\pi p_{1}}{2}\right)+R_{b}^{2}C_{1}^{2}\omega^{2p_{1}}+1}+\frac{\cos\left(\frac{\pi p_{2}}{2}\right)}{C_{2}\omega^{p_{2}}}$$

(8) $$Z_{i}=\frac{R_{b}^{2}C_{1}\omega^{p_{1}}\sin\left(\frac{\pi p_{1}}{2}\right)}{2R_{b}C_{1}\omega^{p_{1}}\cos\left(\frac{\pi p_{1}}{2}\right)+R_{b}^{2}C_{1}^{2}\omega^{2p_{1}}+1}+\frac{\sin\left(\frac{\pi p_{2}}{2}\right)}{C_{2}\omega^{p_{2}}}$$

where *p*_1_ is the deviation semicircle from the vertical axis while *p*_2_ is the deviation of the spike from the horizontal axis. The high and low-frequency capacitances are designated as *C*_1_ and *C*_2_, respectively. For the plot that consists of only a spike (CSMCD3) where *R_b_* and CPE are connected in series, the *Z_r_* and *Z_i_* of the electrolyte can be expressed as:

(9) $$Z_{r}=\frac{\cos\left(\frac{\pi p}{2}\right)}{C\omega^{p}}+R_{b}$$

(10) $$Z_{i}=\frac{\sin\left(\frac{\pi p}{2}\right)}{C\omega^{p}}$$

The obtained circuit element values are listed in Table 2. It is noticed that CPE2 values are increased with the increases in glycerol concentration which explained the enhancement of the number of ions in the electrolytes which increases the availability for electrode polarization, hence increasing the capacitance value at low frequency [56]. These also contribute to better mobility and dissociation of ions which also verifies the formation of attractive forces between polymer chain segments and glycerol molecules that results in a lower cohesive attraction and further enhance the ionic conductivity of the electrolytes [57,58,59]. Marf et al. [60] stated that the mobility of ions and flexibility of the polymer chain as well as the usage of glycerol as a plasticizer are the significant factors that affect the ionic conductivity of the polymer electrolytes.

### 3.2. FTIR Study

**Figure 2:**
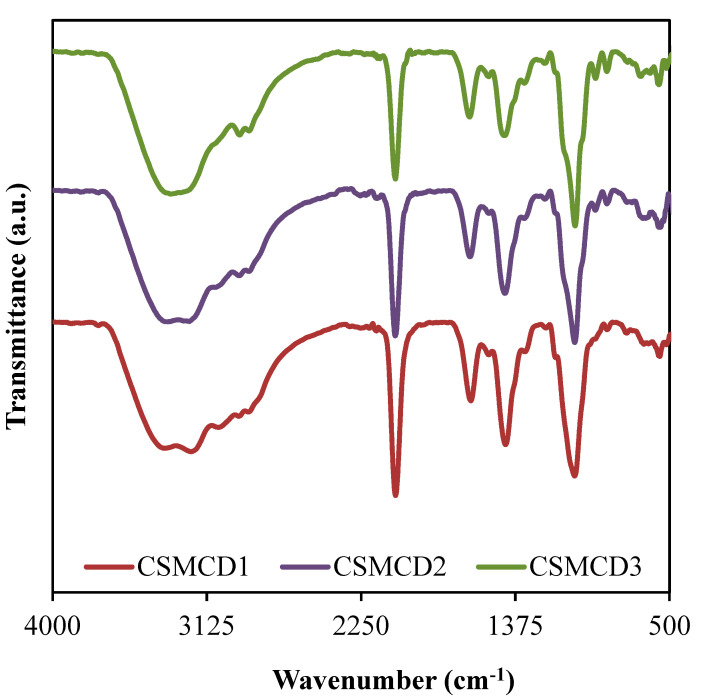
FTIR spectra of the electrolytes.

The FTIR spectra for the electrolytes (as shown in Figure 2) is important to identify the complexation as well as the interaction between the electrolyte components which are polymers (CS and MC), NH_4_SCN salt, and glycerol. The band assignments based on the FTIR spectra are tabulated in Table 3.

The vibrational peak at 3407 cm^−1^ (CSMCD1) corresponds to the O–H stretching, where the high intensity of this peak exposed the well-developed interaction that leads to the increase in ions dissociation which is important in increasing the conductivity [61,62]. As mentioned by Hamsan et al. [63], the indicator of an interaction between two polymers in a polymer blend system can be determined through the peaks shifting of the functional groups containing oxygen atoms such as hydroxyl and ether groups. In the CS–MC–NH4SCN system added with glycerol, the oxygen atoms in polymer form a hydrogen bond with the hydrogen atom of hydroxyl in another polymer [32]. Besides, the O–H bands are also credited to the NH_4_^+^ asymmetry which explains the bending of NH_4_^+^ that could result in a greater tendency for the H^+^ to be released [64]. Moreover, the bands located at 2957 cm^−1^ and 2895 cm^−1^ in CSMCD1 are due to the C–H stretching, symmetrically and asymmetrically [21,65]. It is noticed that the C-H symmetrical stretching is shifted to a lower wavenumber (WN) of 2954 and 2950 cm^−1^ in CSMCD2 and CSMCD3, respectively. Meanwhile, the C-H asymmetrical stretching is observed to shift to a higher WN as the glycerol concentration is increased. This movement of peaks proves the development of complex interaction between the CS–MC–NH4SCN system with plasticizer [66].

Furthermore, the peaks related to the C=O and C=C stretchings can be observed at 1640 and 1441 cm^−1^, respectively [67,68,69]. These peaks are then shifted towards a higher WN when the concentration of glycerol is increased, which explains the interaction between glycerol and cations from the polymer–salt complex [62]. A comparable WN range for these bands was reported by Aziz et al. [67] and Wang et al. [68]. Another obvious sharp peak credited to the C–O stretching is observed at 1044, 1043, and 1039 cm^−1^ for CSMCD1, CSMCD2, and CSMCD3 electrolytes, respectively. As this peak becomes sharper at a high concentration of glycerol, a small shoulder peak that is assigned to C-H_2_ rocking has appeared at 957, 961, and 968 cm^−1^ for CSMCD1, CSMCD2, and CSMCD3 electrolytes, respectively. This further proves the interaction between the CS–MC–NH4SCN and glycerol.

**Figure 3:**
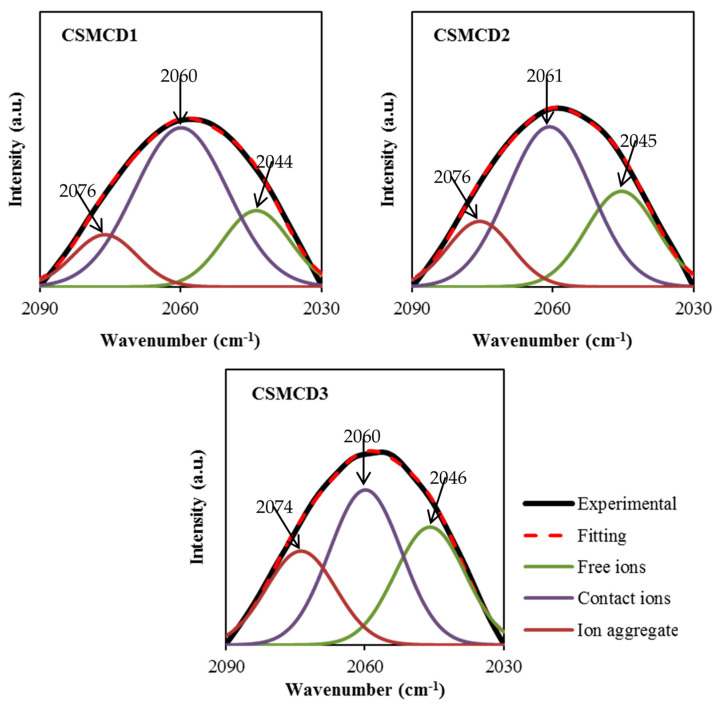
Deconvoluted FTIR spectra at 2030–2090 cm^−1^.

In addition, the FTIR analysis is also beneficial to determine the association and dissociation of ions within the electrolytes [76]. There are two possible reactive sites in the thiocyanate anion (SCN^-^)which can form N-bonding (CN stretching) and S-bonding (CS stretching) as well as the complexes of the N and S atoms (SCN bending) [77]. According to Shamsuri et al. [32], the association and dissociation of ions can be identified in the overlapping spectra between 2030 to 2090 cm^−1^ in the PVA–MC–NH4SCN electrolyte system. Besides, Woo et al. [77] mentioned in their report that the free ions, contact ions pairs, and ion aggregates in the poly(*ε*-caprolactone) incorporated with NH_4_SCN salt were located at 2040, 2058, and 2074 cm^−1^, respectively. In this work, FTIR spectra at 2030–2090 cm^−1^ are selected to be DVN (Figure 3) in order to evaluate the percentage of the area under the peak of each band, which can be calculated using Equation (2). The curves of the FTIR spectra of the SCN^-^ stretching modes in the CS–MC–NH4SCN with a glycerol content ranging from 14 wt.% to 42 wt.% are presented in Figure 3. The DVN of the FTIR spectra is used as an approach technique to separate the overlapping bands accordingly. The increase in free ions in the system suggests that more H^+^ is being dissociated from NH_4_^+^, resulting in a rise in ionic conductivity, whereas the ion pairs represent the ions including NH_4_^+^ or SCN^−^ [78,79].

**Figure 4:**
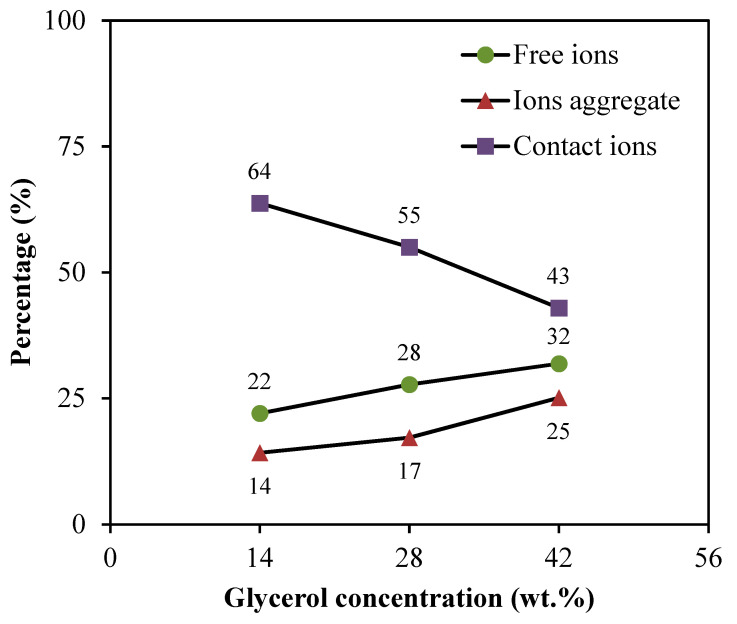
Percentage of free ions, contact ions pairs, and ion aggregates for the electrolytes.

The free ions peak is observed at 2044–2046 cm^−1^, while the contact ions and ion aggregates peaks are located at 2060–2061 cm^−1^ and 2074–2076 cm^−1^, respectively. The percentage of ionic species is determined through the integral area fraction under the curve as summarized in Figure 4. It can be observed that increasing the glycerol concentration induces an increase in the free ions, whereas decreasing leads to a substantial reduction in contact ions. At 14 wt.% of glycerol, contact ions, free ions, and ion aggregates display a percentage of 64%, 22%, and 14%, respectively. As further glycerol was comprised, the integral percentage of free ions increased to (28%) and (32%) at (28 wt.%) and (42 wt.%), correspondingly. However, the integral percentage of ion aggregates was found to be slightly increased. Additionally, the association between different ionic species tends to be clear. Once the percentage of free ions reaches the highest value, the contact ion pairs demonstrate the lowest value. The reduction in contact ions is due to the increment of the concentration of free ions and ion aggregates in the electrolytes. The glycerol concentration is found to impact the percentage of ion species since the intensity of free ions peak becomes higher when the concentration of glycerol increases. This is highly harmonized with the ionic conductivity trend. The percentage of free ions for CSMCD3 is the highest of 32% with the minimum percentage of contact ions and ion aggregates. A comparable observation was reported by Noor and Isa [40] and Brza et al. [80].

Based on the free ions percentage, the transport parameters; number density (*n*), ionic mobility (*μ*), and diffusion coefficient (*D*) can be calculated using Equations (3)–(5), as listed in Table 4. The results illustrate the influence of glycerol concentration on the values of the number density of ions, as well as the ionic mobility and diffusion coefficient. It is noteworthy that the number of ions (*n*) tends to increase steadily as the concentration of glycerol increases. In the meantime, the ionic mobility (*μ*) and diffusion coefficient (*D*) are observed to obey the ionic conductivity trend. Here, it is worth mentioning that the ionic conductivity of the current system is increased by increasing (*n*) value, as revealed by the value of the free ion, which improved progressively by further adding glycerol into the system. The increment of the ionic mobility (*μ*) and diffusion coefficient (*D*) is due to the chain flexibility enhancement caused by the inclusion of glycerol. This is owing to the fact that the incorporation of extra glycerol dissociates extra salts to the free ions, thereby, raising the number of charge carriers in the system. The CSMCD3 electrolyte has the highest values of *n*, *μ* and *D* that optimized at 1.27 × 10^23^ cm^−3^, 1.13 × 10^−8^ cm^2^·V^−1^·s^−1^ and 2.95 × 10^−10^ cm^2^·s^−1^, respectively. The findings obtained from the ionic transport study in this work can conclude that the ionic conductivity result can be affected by the number density values, hence describes the influence of transport parameters on the ionic conductivity results [81,82,83].

### 3.3. Dielectric and Electric Modulus Analyses

**Figure 5:**
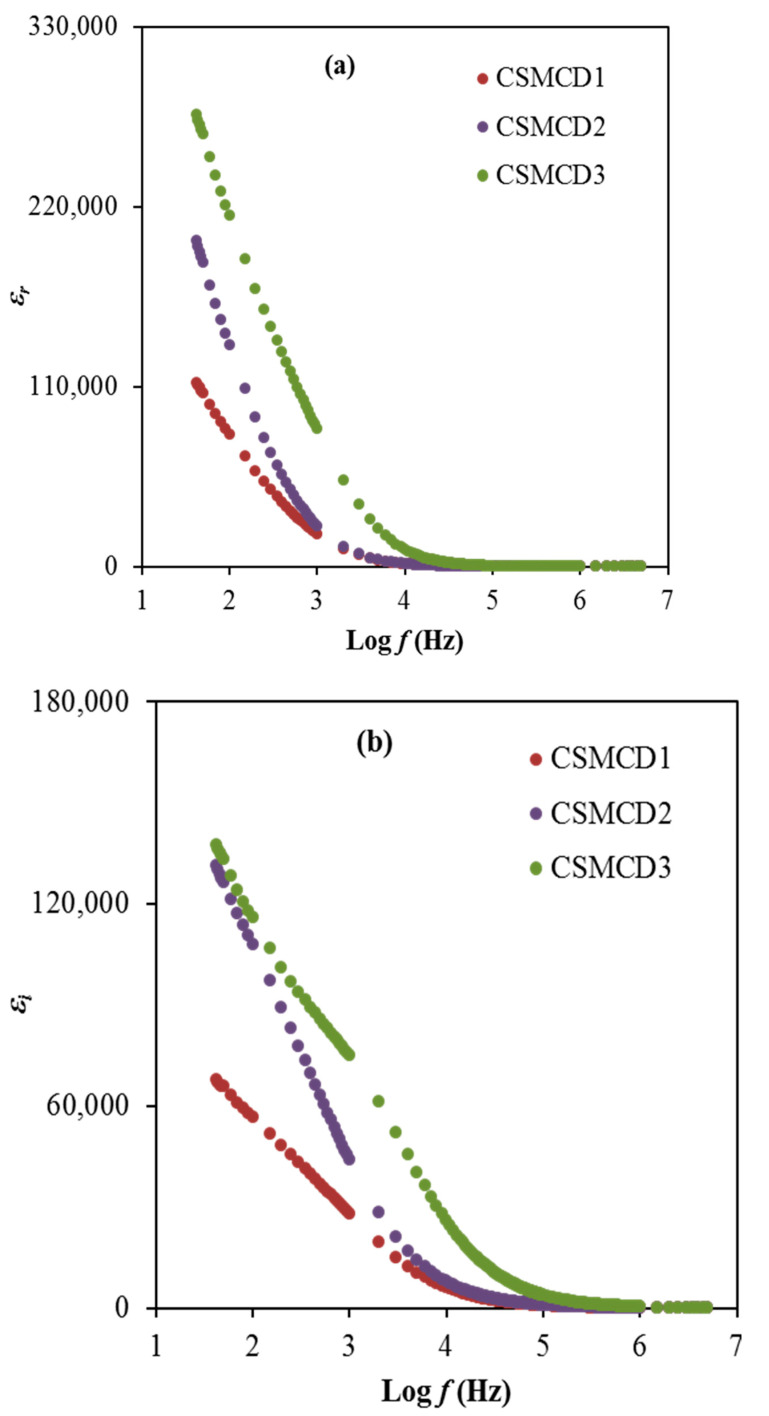
The plot of (**a**) *ɛ_r_* and (**b**) *ɛ_i_* for the electrolytes.

In condensed matter physics, the ion conduction process and dielectric properties in solids are two of the most studied subjects. In particular, dielectric properties in solid polymer-based electrolytes are an effective tool for obtaining knowledge about the electrical properties. The dielectric learning of each electrolyte is helpful for advancing the understanding of the conductivity performance as well as the polarization effects. The dielectric constant, *ɛ_r_* is the amount of charges stored while the dielectric loss, *ɛ_i_* is the energy loss during the movement of ions within the electrolytes. Furthermore, the dielectric constant plays a crucial role in demonstrating the dissolving potential of polymeric materials for a salt. Both dielectric parameters can be expressed by using Equations (11) and (12) and are depicted in Figure 5 [6].

(11) $$\varepsilon_{r}=\frac{Z_{i}}{\omega C_{o}\left(Z_{r}^{2}+Z_{i}^{2}\right)}$$

(12) $$\varepsilon_{i}=\frac{Z_{r}}{\omega C_{o}\left(Z_{r}^{2}+Z_{i}^{2}\right)}$$

where $\;\varepsilon_{r},\;\mathrm{and}\;\varepsilon_{i}$ refer to dielectric constant, and loss, respectively. ω denotes the field applied of angular frequency ω = 2πf. *C_o_* is the capacitance and its equal to ε°A/t, once ε° is the free space permittivity, *A* denotes the electrode area, *t* stands for the electrolyte thickness. It is obviously observed that the highest conducting (highest DC conductivity) electrolyte (CSMCD3) achieves the highest dielectric properties which can be noticed at a lower frequency. The DC conductivity (σ_dc_ = Ʃ*nqμ*) and ε_r_ relation are interrelated qualitatively. The value of *n* (carrier density) is linked with the dissociation energy (U) and ε_r_ which is described using (*n = n_o_exp^(−U/ɛ^_r_^K^_B_^T)^*), where T and K_B_ are the absolute temperatures and Boltzmann constant. Thus any amplification in εr value means an improvement in n and as a result an increase in σdc. The aggregation of charge carriers or the polarization effect near the electrodes results in a large dielectric constant at low frequencies [84,85]. Both dielectric properties values are found to reach approximately constant values at higher frequencies. These reductions are caused by the absence of the diffusion of the excessive ions that leads to the rapid periodic reversal of the electric field [86,87]. Dielectric permittivity decreases with rising frequencies, as the dipoles in the system cannot rotate rapidly, resulting in the lag between the applied field and oscillation of the dipole frequency. This dielectric analysis verifies the non-Debye behavior of the electrolytes in this work.

**Figure 6:**
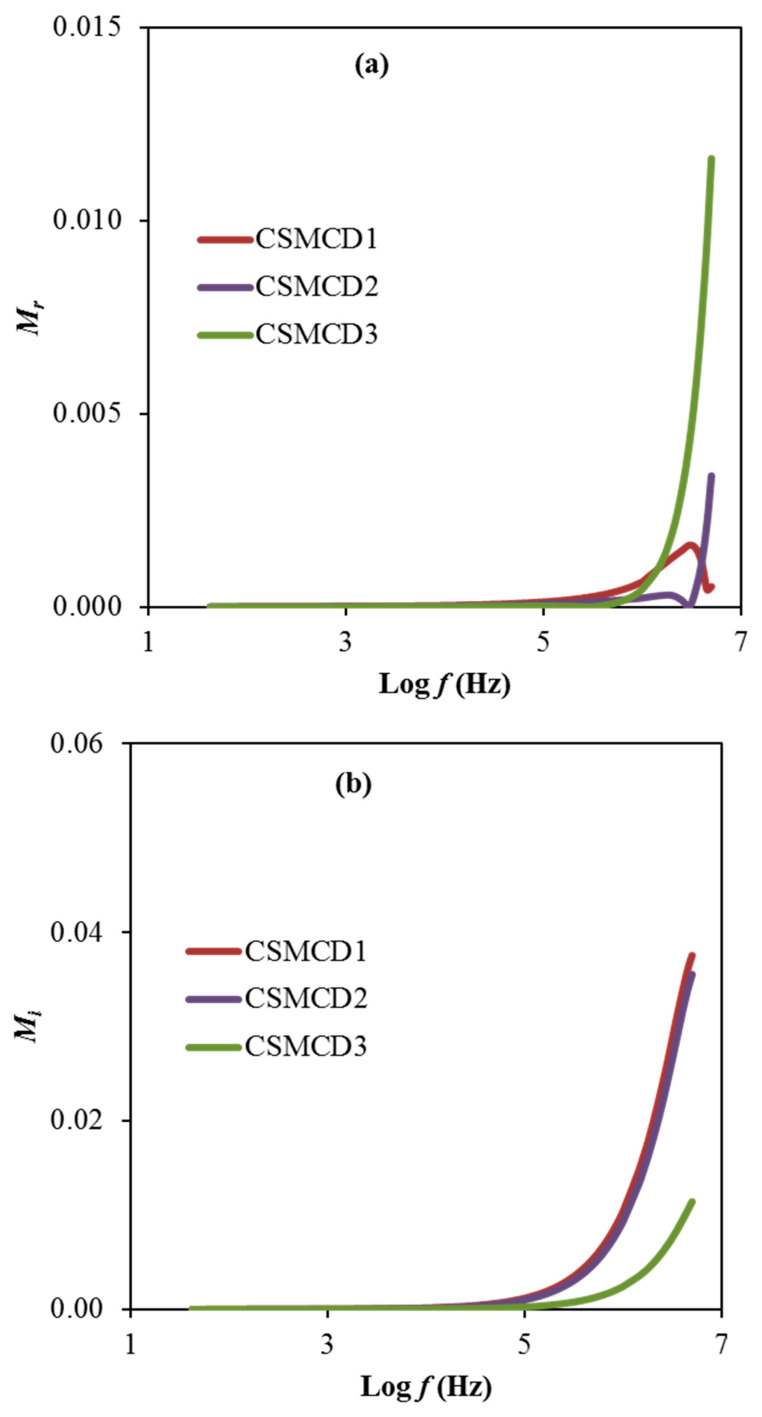
The plot of (**a**) *M_r_* and (**b**) *M_i_* for the electrolytes.

Another noteworthy analysis for the electrolyte is the electric modulus that is examined to support the dielectric results. Figure 6a,b exhibit the real part, *M_r_* and imaginary part, *M_i_* of the electric modulus, respectively calculated using expressions 13 and 14.

(13) $$M_{r}=Z_{i}C_{o}\omega$$

(14) $$M_{i}=Z_{r}C_{o}\omega$$

Based on electrical modulus plots in Figure 6, it is observed the presence of a dominant long tail at a lower frequency explained by the polarization effect of the electrodes that is consequently increased when the frequency increases [2]. However, the CSMCD1 and CSMCD2 electrolytes show a near plot of *M_r_* which might be due to the slight difference of the transport parameters and also the ionic conductivity values. This observation pattern is also reported in the polymer electrolyte studies by Hadi et al. [88] and Ponmani et al. [89]. For the reason of the sudden increase in the *M_r_* and *M_i_* values, the electrolytes in this work are classified as good ionic conductors [90]. *M_r_* and *M_i_* declined with limited tails at the low frequency region, denotation that the electrode polarization will make an insignificant contribution. The electrode polarization is linked to a higher capacitance value. The *M_r_* and *M_i_* spectra are in dissimilar manners in comparison with the prototype of ε_r_ and ε_i_. At low-frequency regions, both ε_r_ and ε_i_ values are found to be higher (see Figure 5), whereas, the *M_r_* and *M_i_* values are decreased. Generally, the *M_r_* and *M_i_* in M* were created as a consequence of the opposition of ε_r_ and ε_i_ in ε*, as it is mathematically formulated in (M* = 1/ε*). The *M_r_* and *M_i_* point to the lowest values at the low frequency region which specify the material capacitive manner [15,85].

### 3.4. Electrochemical Characterizations

**Figure 7:**
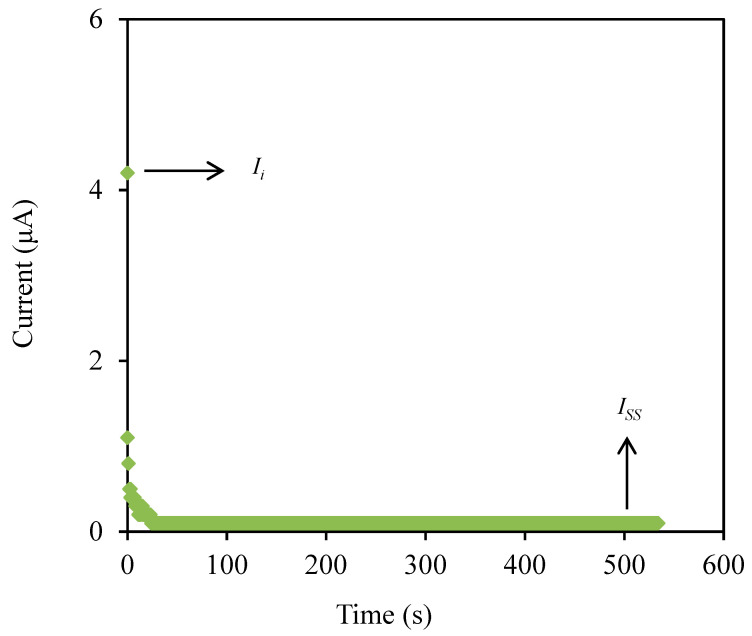
The polarization plot of CSMCD3 electrolyte.

The transference number measurement (TNM) and linear sweep voltammetry (LSV) are useful techniques to determine the contribution of ionic species and also identify the breakdown voltage of the highest conducting electrolyte (CSMCD3) for the application in energy devices. The polarization plot of the CSMCD3 electrolyte is plotted in Figure 7.

**Figure 8:**
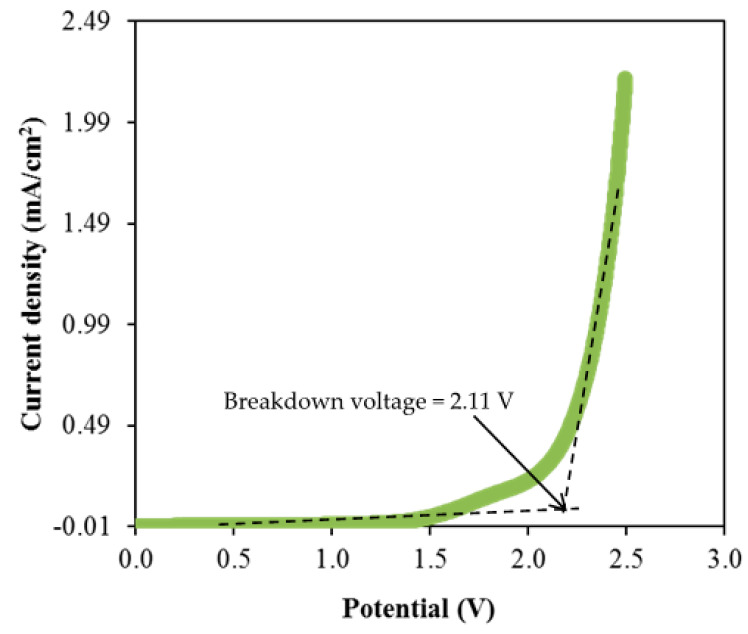
LSV plot of CSMCD3 electrolyte at room temperature.

The CSMCD3 electrolyte exhibits a drastic drop of current in Figure 7 before it is optimized at a steady-state reading. The noteworthy fall is might be due to ions blockage at the electrode surfaces which consequently led to a constant current flow because the ions drifting is equivalent to the diffusion of the ion and causes the electrons to be the only species to pass through [91]. The current steady-state due to electron is obtained when ions are totally reduced in the system [86]. The transference numbers of the ion (*t_ion_*) and electron (*t_e_*) can be calculated using the following equations.

(15) $$t_{ion}=\frac{I_{i}-I_{ss}}{I_{i}}$$

(16) $$t_{e}=\frac{I_{ss}}{I_{i}}$$

where *I_i_* and *I_ss_* represent the current at initial and at steady state, respectively. The *t_ion_* value obtained by CSMCD3 electrolyte is 0.976, while the calculated *t_e_* value is 0.024. The ions will be the dominant charge species if the *t_ion_* value is close to unity [92]. For the comparison, Shukur et al. [27] reported a comparable *t_ion_* value of 0.98 for the CS–NH_4_Br electrolyte system. The CMC–NH4SCN system by Noor and Isa [40] also reported a high *t_ion_* value of 0.93. In our previous work, the highest conducting electrolyte has been tested for TNM analysis and showed a high *t_ion_* value [93]. Furthermore, the electrolyte’s maximum operating voltage can be obtained from the LSV study as plotted in Figure 8 [94].

**Figure 9:**
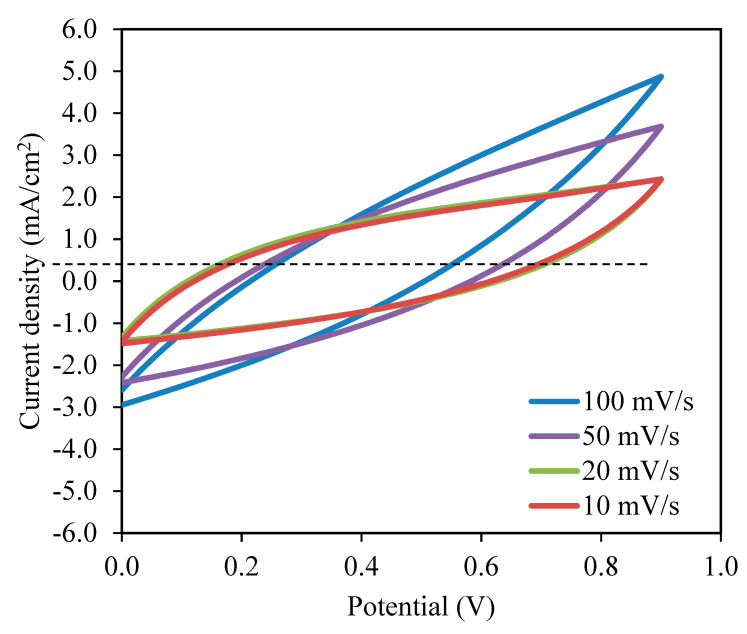
Cyclic voltammetry (CV) curves of the CSMCD3 electrolyte at room temperature.

**Figure 10:**
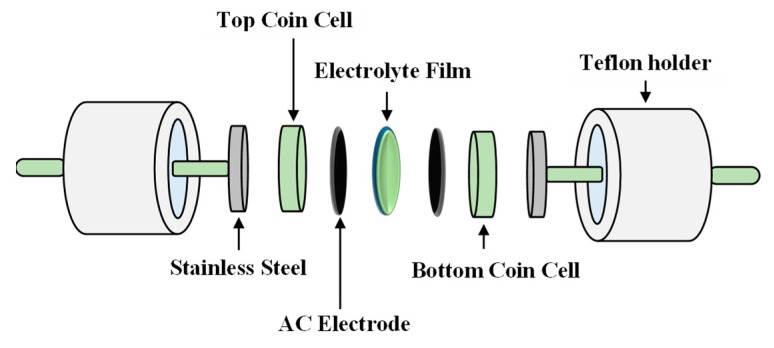
Schematic diagram of the electrochemical double-layer capacitor (EDLC) setup.

As observed in Figure 8, the current density value is unnoticeable until it reaches 2.11 V and no redox reaction is found to occur up to this point within the electrolyte [95]. Then, the current density is noticed to gradually increase from 2.11 V onwards which verifies the breakdown voltage of the electrolyte. The breakdown voltage for the glycerolized corn starch–LiOAc electrolyte system was reported at 2.10 V [96]. Brza et al. [12] studied the PVA–NH4SCN–Cd(II) complex plasticized with glycerol and the breakdown voltage of the system was 2.10 V. The CSMCD3 electrolyte was found to be suitable for future applications since most of the energy devices required a minimum breakdown voltage of 1.0 V [97,98,99]. Therefore, the performance of the CSMCD3 electrolyte is further evaluated in the fabrication EDLC using cyclic voltammetry (CV). Figure 9 depicts the CV curves of the CSMCD3 electrolyte at different scan rates. The schematic diagram of the EDLC cell can be depicted in Figure 10.

There are invisible peaks related to the occurrence of the oxidation or reduction process in the CV plot [100]. No redox peak can be seen in the CV plot, representing the occurence of a charge double-layer at the surface of activated carbon electrodes, that is, capacitive performance [42,48]. The leaf-like shape of the CV curve is observed at a higher scan rate turned to a rectangular-like shape when the scan rates are reduced. The factors that affect these changes could be the porosity of carbon electrodes and the internal resistance presence during the scanning process [101,102]. The non-Faradaic process means the EDLC’s charge storage mechanism depends on ion buildup at the interfaces between electrodes and electrolytes [48,66]. By substituting the initial potential, *V_i_*, and final potential, *V_f_* of 0.0 V and 0.9 V, respectively into Equation (17), the specific capacitance, *C_s_* can be calculated as tabulated in Table 5 [52].

(17) $$C_{s}=\overset{V_{f}}{\underset{V_{i}}{\int }}\frac{I\left(V\right)dV}{2mv\left(V_{2}-V_{1}\right)}$$

where *m* represents the mass of active material while *v* is the scan rate value. The area under the CV curve that is represented by *I(V)dV* was obtained via Origin 9.0 software [103].

The highest *C_s_* value obtained by the EDLC in this work is 98.08 F/g at 10 mV/s. When the scan rates increased to 20, 50, and 100 mV/s, the *C_s_* values were reduced to 51.44, 24.74, and 11.02 F/g, respectively. The shorter contact period for ions and electrodes to full the charge and discharge process at higher scan rates resulted in a lower amount of stored energy. A different situation occurred during lower scan rates where the ions have ample time to be absorbed on the electrolyte surfaces, hence the amount of energy stored is higher [104,105]. The lesser energy loss at lower scan rates resulted in a high amount of charge to be stored [106]. This hypothesis elaborates that the formation of double-layer charge on the electrode surfaces will lead to the storage of potential energy [107]. As the illustration of CV (Figure 9) reveals no apparent reversible hump, it is realistic to terminate that a quick Faradaic reversible feedback has not occurred besides the formation of the double layer [66]. An equivalent outcome of the *C_s_* values is reported in the literature [108,109].

## 4. Conclusions

Three different concentrations of glycerol were successfully incorporated as plasticizers into the CS–MC–NH4SCN electrolyte system. The highest room temperature conductivity of 2.29 × 10^−4^ S cm^−1^ was obtained when 42 wt.% of glycerol (CSMCD3) was introduced to the CS–MC–NH4SCN electrolyte system. The circuit element was further studied by employing the electrical equivalent circuits (EECs). FTIR spectra revealed the complexation and interaction of polymer electrolyte components. The transport parameters were determined from the percentage of free ions obtained from the deconvolution of selected peaks of FTIR spectra. Based on the dielectric analysis, the electrolytes in this work followed non-Debye behavior. The highest conducting electrolyte (CSMCD3) showed the dominancy of ions where the ionic transference number, *t_ion_* value (0.976) was near to infinity with the voltage of breakdown at 2.11 V. The specific capacitance, *C_s_* of the fabricated EDLC was obtained by using cyclic voltammetry (CV) where the *C_s_* values were increased as the scan rates reduced with the highest *C_s_* value of 98.08 F/g at 10 mV/s.

## Figures and Tables

**Table 1:** The *R_b_* and conductivity values of the electrolytes.

Electrolyte	*R_b_* (Ohm)	Conductivity (S cm^−1^)
CSMCD1	290.22	5.31 × 10^−5^
CSMCD2	234.19	6.59 × 10^−5^
CSMCD3	67.37	2.29 × 10^−4^

**Table 2:** The circuit elements values for the electrolytes. CPE: constant phase element.

Electrolyte	*p_1_* (rad)	CPE 1 (F)	*p_2_* (rad)	CPE 2 (F)
CSMCD1	0.46	1.43 × 10^−5^	1.08	4.65 × 10^−6^
CSMCD2	0.49	1.11 × 10^−5^	1.05	7.41 × 10^−6^
CSMCD3	-	-	1.13	8.93 × 10^−6^

**Table 3:** Vibrational assignments of FTIR spectra for the electrolytes.

Wavenumber (cm^−1^)			Assignments	References
CSMCD1	CSMCD2	CSMCD3		
3407	3405	3392	O–H stretching	[67,68,69]
2957	2954	2950	C–H symmetrical stretching	[21,65]
2895	2898	2901	C–H asymmetrical stretching	[21,65]
2059	2062	2063	S–C≡N stretching	[32,33]
1640	1645	1647	C=O stretching	[67,68,69]
1441	1448	1453	C=C stretching	[67,68]
1044	1043	1039	C–O stretching	[67,68,70,71,72]
957	961	968	C–H_2_ rocking	[73,74,75]

**Table 4:** The calculated transport parameters of the electrolytes.

Electrolyte	*n* (cm^−3^)	*μ* (cm^2^V^−1^s^−1^)	*D* (cm^2^s^−1^)
CSMCD1	6.30 × 10^22^	5.26 × 10^−9^	1.37 × 10^−10^
CSMCD2	6.80 × 10^22^	6.05 × 10^−9^	1.58 × 10^−10^
CSMCD3	1.27 × 10^23^	1.13 × 10^−8^	2.95 × 10^−10^

**Table 5:** The Cs values of EDLC at different scan rates.

Scan Rate (mV/s)	Specific Capacitance, *C_s_* (F/g)
10	98.08
20	51.44
50	24.74
100	11.02

## Data Availability

Exclude this statement because the study did not report any data.

## References

[1] Yusof Y.M., Shukur M.F., Illias H.A., Kadir M.F.Z. (2014). Conductivity and electrical properties of corn starch-chitosan blend biopolymer electrolyte incorporated with ammonium iodide. Phys. Scr..

[2] Kadir M., Hamsan M. (2018). Green electrolytes based on dextran-chitosan blend and the effect of NH_4_SCN as proton provider on the electrical response studies. Ionics.

[3] Fadzallah I.A., Noor I.M., Careem M.A., Arof A.K. (2016). Investigation of transport properties of chitosan-based electrolytes utilizing impedance spectroscopy. Ionics.

[4] Prokhorov E., Luna-Bárcenas G., González-Campos J.B., Kovalenko Y., García-Carvajal Z.Y., Mota-Morales J. (2016). Proton conductivity and relaxation properties of chitosan-acetate films. Electrochim. Acta.

[5] Ibnu M., Ahmad H., Ikmar M., Mohamad N. (2020). Natural Inspired Carboxymethyl Cellulose (CMC) Doped with Ammonium Carbonate (AC) as Biopolymer Electrolyte. Polymers.

[6] Mohamed A.S., Shukur M.F., Kadir M.F.Z., Yusof Y.M. (2020). Ion conduction in chitosan-starch blend based polymer electrolyte with ammonium thiocyanate as charge provider. J. Polym. Res..

[7] Inagaki M., Konno H., Tanaike O. (2010). Carbon materials for electrochemical capacitors. J. Power Source.

[8] Zhang D., Zhang X., Chen Y., Yu P., Wang C., Ma Y. (2011). Enhanced capacitance and rate capability of graphene/polypyrrole composite as electrode material for supercapacitors. J. Power Source.

[9] Pell W.G., Conway B.E. (2004). Peculiarities and requirements of asymmetric capacitor devices based on combination of capacitor and battery-type electrodes. J. Power Source.

[10] Liew C.W., Ramesh S., Arof A.K. (2016). Enhanced capacitance of EDLCs (electrical double layer capacitors) based on ionic liquid-added polymer electrolytes. Energy.

[11] Wang H., Lin J., Shen Z.X. (2016). Polyaniline (PANi) based electrode materials for energy storage and conversion. J. Sci. Adv. Mater. Devices.

[12] Brza M.A., Aziz S.B., Anuar H., Dannoun E.M.A., Ali F., Abdulwahid R.T., Al-Zangana S., Kadir M.F.Z. (2020). The Study of EDLC Device with High Electrochemical Performance Fabricated from Proton Ion Conducting PVA-Based Polymer Composite Electrolytes Plasticized with Glycerol. Polymers.

[13] Samsudin A., Isa M. (2012). Structural and electrical properties of carboxy methylcellulose-dodecyltrimethyl ammonium bromide-based biopolymer electrolytes system. Int. J. Polym. Mater. Polym. Biomater..

[14] Samsudin A.S., Isa M.I.N. (2012). Ion Conducting Mechanism of Carboxy Methylcellulose Doped With Ionic Dopant Salicylic Acid Based Solid Polymer Electrolytes. Int. J. Appl. Sci. Technol..

[15] Aziz S.B., Hamsan M.H., Kadir M.F.Z., Woo H.J. (2020). Design of Polymer Blends Based on Chitosan: POZ with Improved Dielectric Constant for Application in Polymer Electrolytes and Flexible Electronics. Adv. Polym. Technol..

[16] Stepniak I., Galinski M., Nowacki K., Wysokowski M., Jakubowska P., Bazhenov V.V., Leisegang T., Ehrlich H., Jesionowski T. (2016). A novel chitosan/sponge chitin origin material as a membrane for supercapacitors-preparation and characterization. RSC Adv..

[17] Hamsan M.H., Aziz S.B., Nofal M.M., Brza M.A., Abdulwahid R.T., Hadi J.M., Karim W.O., Kadir M.F.Z. (2020). Characteristics of EDLC device fabricated from plasticized chitosan:MgCl_2_ based polymer electrolyte. J. Mater. Res. Technol..

[18] Zulkifli A.M., Said N.I.A.M., Aziz S.B., Hisham S., Shah S., Bakar A.A., Abidin Z.H.Z., Tajuddin H.A., Sulaiman L., Brza M.A. (2020). Electrochemical characteristics of phthaloyl chitosan based gel polymer electrolyte for dye sensitized solar cell application. Int. J. Electrochem. Sci..

[19] Khiar A.S.A., Puteh R., Arof A.K. (2006). Characterizations of chitosan-ammonium triflate (NH_4_CF_3_SO_3_) complexes by FTIR and impedance spectroscopy. Phys. Status Solidi Appl. Mater. Sci..

[20] Hadi J.M., Aziz S.B., Nofal M.M., Hussen S.A., Hamsan M.H., Brza M.A., Abdulwahid R.T., Kadir M.F.Z., Woo H.J. (2020). Electrical, dielectric property and electrochemical performances of plasticized silver ion-conducting chitosan-based polymer nanocomposites. Membranes.

[21] Ndruru S.T.C.L., Wahyuningrum D., Bundjali B., Arcana I.M. (2020). Preparation and characterization of biopolymer electrolyte membranes based on LiClO_4_-complexed methyl cellulose as lithium-ion battery separator. J. Eng. Technol. Sci..

[22] Samsudin A.S., Kuan E.C.H., Isa M.I.N. (2011). Investigation of the potential of proton-conducting biopolymer electrolytes based methyl cellulose-glycolic acid. Int. J. Polym. Anal. Charact..

[23] García M.A., Pinotti A., Martino M., Zaritzky N. (2009). Electrically treated composite FILMS based on chitosan and methylcellulose blends. Food Hydrocoll..

[24] Selvalakshmi S., Vijaya N., Selvasekarapandian S., Premalatha M. (2017). Biopolymer agar-agar doped with NH_4_SCN as solid polymer electrolyte for electrochemical cell application. J. Appl. Polym. Sci..

[25] Saadiah M.A., Tan H.M., Samsudin A.S. (2020). Enhancement of proton conduction in carboxymethyl cellulose-polyvinyl alcohol employing polyethylene glycol as a plasticizer. Bull. Mater. Sci..

[26] Hamsan M.H., Shukur M.F., Aziz S.B., Kadir M.F.Z. (2019). Dextran from Leuconostoc mesenteroides-doped ammonium salt-based green polymer electrolyte. Bull. Mater. Sci..

[27] Shukur M.F., Hamsan M.H., Kadir M.F.Z. (2019). Investigation of plasticized ionic conductor based on chitosan and ammonium bromide for EDLC application. Mater. Today Proc..

[28] Asnawi A.S.F.M., Hamsan M.H., Kadir M.F.Z., Aziz S.B., Yusof Y.M. (2020). Investigation on electrochemical characteristics of maltodextrin–methyl cellulose electrolytes. Mol. Cryst. Liq. Cryst..

[29] Min I. (2014). Conductivity study of Carboxyl methyl cellulose Solid biopolymer electrolytes (SBE) doped with Ammonium Fluoride. Res. J. Recent Sci..

[30] Moniha V., Alagar M., Selvasekarapandian S., Sundaresan B., Hemalatha R., Boopathi G. (2018). Synthesis and characterization of bio-polymer electrolyte based on iota-carrageenan with ammonium thiocyanate and its applications. J. Solid State Electrochem..

[31] Aziz S.B., Hamsan M.H., Karim W.O., Kadir M.F.Z., Brza M.A., Abdullah O.G. (2019). High Proton Conducting Polymer Blend Electrolytes Based on Chitosan:Dextran with Constant Specific Capacitance and Energy Density. Biomolecules.

[32] Shamsuri N.A., Zaine S.N.A., Yusof Y.M., Yahya W.Z.N., Shukur M.F. (2020). Effect of ammonium thiocyanate on ionic conductivity and thermal properties of polyvinyl alcohol–methylcellulose–based polymer electrolytes. Ionics.

[33] Hemalatha R., Alagar M., Selvasekarapandian S., Sundaresan B., Moniha V. (2019). Studies of proton conducting polymer electrolyte based on PVA, amino acid proline and NH4SCN. J. Sci. Adv. Mater. Devices.

[34] Liang S., Huang Q., Liu L., Yam K.I. (2009). Microstructure and molecular interaction in glycerol plasticized chitosan/poly(vinyl alcohol) blending films. Macromol. Chem. Phys..

[35] Yusof Y.M., Kadir M.F.Z. (2016). Electrochemical characterizations and the effect of glycerol in biopolymer electrolytes based on methylcellulose-potato starch blend. Mol. Cryst. Liq. Cryst..

[36] Kadir M., Salleh N., Hamsan M., Aspanut Z., Majid N., Shukur M. (2018). Biopolymeric electrolyte based on glycerolized methyl cellulose with NH4Br as proton source and potential application in EDLC. Ionics.

[37] Asnawi A.S.F.M., Aziz S.B., Nofal M., Abdulwahid R.T., Kadir M.F.Z., Hamsan M.H., Brza M.A., Yusof Y.M., Abdilwahid R.T. (2020). Glycerolized Li^+^ Ion Conducting Chitosan-Based Polymer Electrolyte for Energy Storage EDLC Device Applications with Relatively High Energy Density. Polymers.

[38] Hamsan H.M., Aziz S., Kadir M.F.Z., Brza M.A., Karim W. (2020). The study of EDLC device fabricated from plasticized magnesium ion conducting chitosan based polymer electrolyte. Polym. Test..

[39] Mustafa M.S., Ghareeb H.O., Aziz S.B., Brza M.A., Al-zangana S., Hadi J.M., Kadir M.F.Z. (2020). Electrochemical characteristics of glycerolized PEO-based polymer electrolytes. Membranes.

[40] Noor N., Isa M. (2019). Investigation on transport and thermal studies of solid polymer electrolyte based on carboxymethyl cellulose doped ammonium thiocyanate for potential application in electrochemical devices. Int. J. Hydrogen Energy.

[41] Ramlli M.A., Isa M.I.N. (2016). Structural and ionic transport properties of protonic conducting solid biopolymer electrolytes based on carboxymethyl cellulose doped with ammonium fluoride. J. Phys. Chem. B.

[42] Aziz S.B., Brza M.A., Hamsan H.M., Kadir M.F.Z., Abdulwahid R.T. (2020). Electrochemical characteristics of solid state double-layer capacitor constructed from proton conducting chitosan-based polymer blend electrolytes. Polym. Bull..

[43] Dannoun E.M.A., Aziz S.B., Brza M.A., Nofal M.M., Asnawi A.S.F.M., Yusof Y.M., Al-Zangana S., Hamsan M.H., Kadir M.F.Z., Woo H.J. (2020). The Study of Plasticized Solid Polymer Blend Electrolytes Based on Natural Polymers and Their Application for Energy Storage EDLC Devices. Polymers.

[44] Kumar M., Tiwari T., Chauhan J.K., Srivastava N. (2014). Erratum: Understanding the ion dynamics and relaxation behavior from impedance spectroscopy of NaI doped Zwitterionic polymer system. Mater. Res. Express.

[45] Aziz S.B. (2018). The Mixed Contribution of Ionic and Electronic Carriers to Conductivity in Chitosan Based Solid Electrolytes Mediated by CuNt Salt. J. Inorg. Organomet. Polym. Mater..

[46] Ramaswamy M., Malayandi T., Subramanian S., Srinivasalu J., Rangaswamy M., Soundararajan V. (2017). Development and Study of Solid Polymer Electrolyte Based on Polyvinyl Alcohol: Mg(ClO_4_)_2_. Polym. Plast. Technol. Eng..

[47] Ramya C.S., Selvasekarapandian S., Savitha T., Hirankumar G., Baskaran R., Bhuvaneswari M.S., Angelo P.C. (2006). Conductivity and thermal behavior of proton conducting polymer electrolyte based on poly (N-vinyl pyrrolidone). Eur. Polym. J..

[48] Aziz S.B., Hamsan M.H., Abdullah R.M., Kadir M.F.Z. (2019). A promising polymer blend electrolytes based on chitosan: Methyl cellulose for EDLC application with high specific capacitance and energy density. Molecules.

[49] Asnawi A.S.F.M., Aziz S.B., Nofal M.M., Yusof Y.M., Brevik I. (2020). Metal Complex as a Novel Approach to Enhance the Amorphous Phase and Improve the EDLC Performance of Plasticized Proton Conducting Chitosan-Based Polymer Electrolyte. Membranes.

[50] Aziz S.B., Hamsan M.H., Kadir M.F.Z., Karim W.O., Abdullah R.M. (2019). Development of polymer blend electrolyte membranes based on chitosan: Dextran with high ion transport properties for EDLC application. Int. J. Mol. Sci..

[51] Hamsan M.H., Aziz S.B., Azha M.A.S., Azli A.A., Shukur M.F., Yusof Y.M., Muzakir S.K., Manan N.S.A., Kadir M.F.Z. (2019). Solid-state double layer capacitors and protonic cell fabricated with dextran from *Leuconostoc mesenteroides* based green polymer electrolyte. Mater. Chem. Phys..

[52] Hamsan M., Shukur M., Aziz S.B., Yusof Y., Kadir M. (2020). Influence of NH_4_Br as an ionic source on the structural/electrical properties of dextran-based biopolymer electrolytes and EDLC application. Bull. Mater. Sci..

[53] Hadi J.M., Aziz S.B., Saeed S.R., Brza M.A., Abdulwahid R.T., Hamsan M.H., Abdullah R.M., Kadir M.F.Z., Muzakir S.K. (2020). Investigation of ion transport parameters and electrochemical performance of plasticized biocompatible chitosan-based proton conducting polymer composite electrolytes. Membranes.

[54] Teo L.P., Buraidah M.H., Nor A.F.M., Majid S.R. (2012). Conductivity and dielectric studies of Li_2_SnO_3_. Ionics.

[55] Aziz S.B., Marif R.B., Brza M.A., Hamsan M.H., Kadir M.F.Z. (2019). Employing of Trukhan model to estimate ion transport parameters in PVA based solid polymer electrolyte. Polymers.

[56] Awasthi P., Das S. (2019). Reduced electrode polarization at electrode and analyte interface in impedance spectroscopy using carbon paste and paper. Rev. Sci. Instrum..

[57] Khiar A.S.A., Anuar M.R.S., Md Parid M.A. (2016). Effect of 1-ethyl-3-methylimidazolium nitrate on the electrical properties of starch/chitosan blend polymer electrolyte. Mater. Sci. Forum.

[58] Yahya M.Z.A., Arof A.K. (2003). Effect of oleic acid plasticizer on chitosan-lithium acetate solid polymer electrolytes. Eur. Polym. J..

[59] Lee D.K., Allcock H.R. (2010). The effects of cations and anions on the ionic conductivity of poly[bis(2-(2-methoxyethoxy)ethoxy)phosphazene] doped with lithium and magnesium salts of trifluoromethanesulfonate and bis(trifluoromethanesulfonyl)imidate. Solid State Ion..

[60] Marf A.S., Aziz S.B., Abdullah R.M. (2020). Plasticized H^+^ ion-conducting PVA:CS-based polymer blend electrolytes for energy storage EDLC application. J. Mater. Sci. Mater. Electron..

[61] Salleh N.S., Aziz S.B., Aspanut Z., Kadir M.F.Z. (2016). Electrical impedance and conduction mechanism analysis of biopolymer electrolytes based on methyl cellulose doped with ammonium iodide. Ionics.

[62] Amran N.N.A., Manan N.S.A., Kadir M.F.Z. (2016). The effect of LiCF_3_SO_3_ on the complexation with potato starch-chitosan blend polymer electrolytes. Ionics.

[63] Hamsan M.H., Shukur M.F., Kadir M.F.Z. (2017). The effect of NH4NO3 towards the conductivity enhancement and electrical behavior in methyl cellulose-starch blend based ionic conductors. Ionics.

[64] Bourahla S., Ali Benamara A., Kouadri Moustefai S. (2014). Infrared spectra of inorganic aerosols: Ab initio study of (NH_4_)_2_SO_4_, NH_4_NO_3_, and NaNO_3_. Can. J. Phys..

[65] Hafiza M.N., Isa M.I.N. (2020). Correlation between structural, ion transport and ionic conductivity of plasticized 2-hydroxyethyl cellulose based solid biopolymer electrolyte. J. Memb. Sci..

[66] Aziz S.B., Hamsan M.H., Brza M.A., Kadir M.F.Z., Abdulwahid R.T., Ghareeb H.O., Woo H.J. (2019). Fabrication of energy storage EDLC device based on CS:PEO polymer blend electrolytes with high Li^+^ ion transference number. Results Phys..

[67] Aziz S.B., Hamsan M.H., Abdullah R.M., Abdulwahid R.T., Brza M.A. (2020). Protonic EDLC cell based on chitosan (CS): Methylcellulose (MC) solid polymer blend electrolytes. Ionics.

[68] Wang H., Liao Y., Wu A., Li B., Qian J., Ding F. (2019). Effect of sodium trimetaphosphate on chitosan-methylcellulose composite films: Physicochemical properties and food packaging application. Polymers.

[69] Salman Y.A.K., Abdullah O.G., Hanna R.R., Aziz S.B. (2018). Conductivity and electrical properties of chitosan-methylcellulose blend biopolymer electrolyte incorporated with lithium tetrafluoroborate. Int. J. Electrochem. Sci..

[70] Mejenom A.A., Hafiza M.N., Isa M.I.N. (2018). X-ray diffraction and infrared spectroscopic analysis of solid biopolymer electrolytes based on dual blend carboxymethyl cellulose-chitosan doped with ammonium bromide. ASM Sci. J..

[71] Poy S.Y., Bashir S., Omar F.S., Saidi N.M., Farhana N.K., Sundararajan V., Ramesh K., Ramesh S. (2020). Poly (1-vinylpyrrolidone-co-vinyl acetate) (PVP-co-VAc) based gel polymer electrolytes for electric double layer capacitors (EDLC). J. Polym. Res..

[72] Misenan M., Isa M., Khiar A. (2018). Electrical and structural studies of polymer electrolyte based on chitosan/ methyl cellulose blend doped with BMIMTFSI. Mater. Res. Express.

[73] Ni’Mah Y.L., Cheng M.Y., Cheng J.H., Rick J., Hwang B.J. (2015). Solid-state polymer nanocomposite electrolyte of TiO_2_/PEO/NaClO_4_ for sodium ion batteries. J. Power Source.

[74] Sownthari K., Suthanthiraraj S.A. (2013). Synthesis and characterization of an electrolyte system based on a biodegradable polymer. Express Polym. Lett..

[75] Asnawi A.S.F.M., Azli A., Hamsan M., Kadir M., Yusof Y. (2020). Electrical and Infrared Spectroscopic Analysis of Solid Polymer Electrolyte Based on Polyethylene Oxide and Graphene Oxide Blend. Malays. J. Anal. Sci..

[76] Rahman N.A.A., Navaratnam S., Abidin S.Z.Z., Latif F.A. (2018). FTIR study on the effect of free ions in PMMA/ENR 50 polymer electrolyte system. AIP Conf. Proc..

[77] Woo H.J., Majid S.R., Arof A.K. (2011). Conduction and thermal properties of a proton conducting polymer electrolyte based on poly (ε-caprolactone). Solid State Ion..

[78] Rani M.S.A., Mohamed N.S., Isa M.I.N. (2015). Investigation of the Ionic Conduction Mechanism in Carboxymethyl Cellulose/Chitosan Biopolymer Blend Electrolyte Impregnated with Ammonium Nitrate. Int. J. Polym. Anal. Charact..

[79] Rasali N.M.J., Nagao Y., Samsudin A.S. (2019). Enhancement on amorphous phase in solid biopolymer electrolyte based alginate doped NH_4_NO_3_. Ionics.

[80] Brza M.A., Aziz S.B., Anuar H., Ali F. (2020). Structural, ion transport parameter and electrochemical properties of plasticized polymer composite electrolyte based on PVA: A novel approach to fabricate high performance EDLC devices. Polym. Test..

[81] Aniskari N.A.B., Mohd Isa M.I.N. (2017). The effect of ionic charge carriers in 2-hydroxyethyl cellulose solid biopolymer electrolytes doped glycolic acid via FTIR-deconvolution technique. J. Sustain. Sci. Manag..

[82] Arya A., Sharma A.L. (2019). Dielectric relaxations and transport properties parameter analysis of novel blended solid polymer electrolyte for sodium-ion rechargeable batteries. J. Mater. Sci..

[83] Aziz S.B., Dannoun E.M.A., Hamsan M.H., Ghareeb H.O., Nofal M.M., Karim W.O., Asnawi A.S.F.M., Hadi J.M., Kadir M.F.Z.A. (2021). A Polymer Blend Electrolyte Based on CS with Enhanced Ion Transport and Electrochemical Properties for Electrical Double Layer Capacitor Applications. Polymers.

[84] Rani M.S.A., Ahmad A., Mohamed N.S. (2018). A comprehensive investigation on electrical characterization and ionic transport properties of cellulose derivative from kenaf fibre-based biopolymer electrolytes. Polym. Bull..

[85] Nofal M.M., Aziz S.B., Hadi J.M., Abdulwahid R.T., Dannoun E.M.A., Marif A.S., Al-Zangana S., Zafar Q., Brza M.A., Kadir M.F.Z. (2020). Synthesis of porous proton ion conducting solid polymer blend electrolytes based on PVA: CS polymers: Structural, morphological and electrochemical properties. Materials.

[86] Ramly K., Isa M.I.N., Khiar A.S.A. (2011). Conductivity and dielectric behaviour studies of starch/PEO+*x* wt-%NH_4_NO_3_ polymer electrolyte. Mater. Res. Innov..

[87] Hadi J.M., Aziz S.B., Mustafa M.S., Brza M.A., Hamsan M.H., Kadir M.F.Z., Ghareeb H.O., Hussein S.A. (2020). Electrochemical impedance study of proton conducting polymer electrolytes based on PVC doped with thiocyanate and plasticized with glycerol. Int. J. Electrochem. Sci..

[88] Hadi J.M., Aziz S.B., Mustafa M.S., Hamsan M.H., Abdulwahid R.T., Kadir M.F.Z., Ghareeb H.O. (2020). Role of nano-capacitor on dielectric constant enhancement in PEO:NH_4_SCN:xCeO_2_ polymer nano-composites: Electrical and electrochemical properties. J. Mater. Res. Technol..

[89] Ponmani S., Kalaiselvimary J., Ramesh Prabhu M. (2018). Structural, electrical, and electrochemical properties of poly(vinylidene fluoride-co-hexaflouropropylene)/poly(vinyl acetate)-based polymer blend electrolytes for rechargeable magnesium ion batteries. J. Solid State Electrochem..

[90] Fuzlin A.F., Rasali N.M.J., Samsudin A.S. (2018). Effect on Ammonium Bromide in dielectric behavior based Alginate Solid Biopolymer electrolytes. IOP Conf. Ser. Mater. Sci. Eng..

[91] Rani M.S.A., Ahmad A., Mohamed N.S. (2018). Influence of nano-sized fumed silica on physicochemical and electrochemical properties of cellulose derivatives-ionic liquid biopolymer electrolytes. Ionics.

[92] Aziz S.B., Hadi J.M., Elham E.M., Abdulwahid R.T., Saeed S.R., Marf A.S., Karim W.O., Kadir M.F.Z. (2020). The study of plasticized amorphous biopolymer blend electrolytes based on polyvinyl alcohol (PVA): Chitosan with high ion conductivity for energy storage electrical double-layer capacitors (EDLC) device application. Polymers.

[93] Chai M.N., Isa M.I.N. (2016). Novel Proton Conducting Solid Bio-polymer Electrolytes Based on Carboxymethyl Cellulose Doped with Oleic Acid and Plasticized with Glycerol. Sci. Rep..

[94] Asnawi A.S.F.M., Aziz S.B., Saeed S.R., Yusof Y.M., Abdulwahid R.T., Al-zangana S., Karim W.O., Kadir M.F.Z. (2020). Solid-State EDLC Device Based on Magnesium Ion-Conducting Biopolymer Composite Membrane Electrolytes: Impedance, Circuit Modeling, Dielectric Properties and Electrochemical Characteristics. Membranes.

[95] Francis K.A., Liew C.W., Ramesh S., Ramesh K., Ramesh S. (2016). Ionic liquid enhanced magnesium-based polymer electrolytes for electrical double-layer capacitors. Ionics.

[96] Sampathkumar L., Christopher Selvin P., Selvasekarapandian S., Perumal P., Chitra R., Muthukrishnan M. (2019). Synthesis and characterization of biopolymer electrolyte based on tamarind seed polysaccharide, lithium perchlorate and ethylene carbonate for electrochemical applications. Ionics.

[97] Shukur M.F., Ithnin R., Kadir M.F.Z. (2014). Electrical characterization of corn starch-LiOAc electrolytes and application in electrochemical double layer capacitor. Electrochim. Acta.

[98] Shuhaimi N.E.A., Alias N.A., Majid S.R., Arof A.K. (2009). Electrical Double Layer Capacitor with Proton Conducting Κ-Carrageenan–Chitosan Electrolytes. Funct. Mater. Lett..

[99] Mazuki N., Abdul Majeed A.P.P., Samsudin A.S. (2020). Study on electrochemical properties of CMC-PVA doped NH_4_Br based solid polymer electrolytes system as application for EDLC. J. Polym. Res..

[100] Kadir M.F.Z., Arof A.K. (2013). Application of PVA-chitosan blend polymer electrolyte membrane in electrical double layer capacitor. Mater. Res. Innov..

[101] Bandaranayake C.M., Weerasinghe W.A.D.S.S., Vidanapathirana K.P., Perera K.S. (2016). A Cyclic Voltammetry study of a gel polymer electrolyte based redox-capacitor. Sri Lankan J. Phys..

[102] Aziz S.B., Brza M.A., Dannoun E.M.A., Hamsan M.H., Hadi J.M., Kadir M.F.Z., Abdulwahid R.T. (2020). The study of electrical and electrochemical properties of magnesium ion conducting CS: PVA based polymer blend electrolytes: Role of lattice energy of magnesium salts on EDLC performance. Molecules.

[103] Lee J.S.M., Briggs M.E., Hu C.C., Cooper A.I. (2018). Controlling electric double-layer capacitance and pseudocapacitance in heteroatom-doped carbons derived from hypercrosslinked microporous polymers. Nano Energy.

[104] Nadiah N.S., Omar F.S., Numan A., Mahipal Y.K., Ramesh S., Ramesh K. (2017). Influence of acrylic acid on ethylene carbonate/dimethyl carbonate based liquid electrolyte and its supercapacitor application. Int. J. Hydrogen Energy.

[105] Chong M.Y., Liew C.W., Numan A., Yugal K., Ramesh K., Ng H.M., Chong T.V., Ramesh S. (2016). Effects of ionic liquid on the hydroxylpropylmethyl cellulose (HPMC) solid polymer electrolyte. Ionics.

[106] Muchakayala R., Song S., Wang J., Fan Y., Bengeppagari M., Chen J., Tan M. (2018). Development and supercapacitor application of ionic liquid-incorporated gel polymer electrolyte films. J. Ind. Eng. Chem..

[107] Fattah N.F.A., Ng H.M., Mahipal Y.K., Numan A., Ramesh S., Ramesh K. (2016). An approach to solid-state electrical double layer capacitors fabricated with graphene oxide-doped, ionic liquid-based solid copolymer electrolytes. Materials.

[108] Syahidah S.N., Majid S.R. (2015). Ionic liquid-based polymer gel electrolytes for symmetrical solid-state electrical double layer capacitor operated at different operating voltages. Electrochim. Acta.

[109] Wang J., Chen G., Song S. (2020). Na-ion conducting gel polymer membrane for flexible supercapacitor application. Electrochim. Acta.

